# *Bmal1* Regulates Coagulation Factor Biosynthesis in Mouse Liver in *Streptococcus oralis* Infection

**DOI:** 10.3389/fcimb.2020.530190

**Published:** 2020-09-16

**Authors:** Lili Chen, Shue Li, Jiaming Nie, Jiajia Zhao, Shaoling Yu, Yaoxu Li, Jinfeng Peng

**Affiliations:** ^1^Department of Stomatology, Union Hospital, Tongji Medical College, Huazhong University of Science and Technology, Wuhan, China; ^2^Hubei Key Laboratory of Oral and Craniomaxillofacial Development and Regeneration, Union Hospital, Tongji Medical College, Huazhong University of Science and Technology, Wuhan, China

**Keywords:** *S. oralis*, bmal1, FVII, FXII, coagulation factor biosynthesis

## Abstract

*Streptococcus oralis (S. oralis*) has been recognized as a fatal pathogen to cause multiorgan failure by contributing to the formation of microthrombus. Coagulation and fibrinolysis systems have been found under the control of circadian clock genes. This study aimed to explore the correlation between *BMAL1* and coagulation factor biosynthesis in *S. oralis* infection. Mice were administered *S. oralis* to induce sepsis, and HepG2 cells were also infected by *S. oralis*. The expression of *BMAL1* of hepatocytes was downregulated in the *S. oralis* infection group, leading to the downregulation of coagulation factor VII (FVII) and the upregulation of the coagulation factor XII (FXII) *in vitro* and *in vivo*. Furthermore, we confirmed that the deficiency of *BAML1* contributed to the elevation of FVII and the decline in FXII by constructing BMAL1-deficiency (*Bmal1*^−/−^) mice. The current result showed that BMAL1 regulates FVII directly. Thus, a novel insight into the coagulation abnormality in *S. oralis* infection was gained that may optimize the treatment of sepsis by rescuing the expression of BMAL1 in the liver.

## Introduction

*Streptococcus oralis* (*S. oralis*) is an oral biofilm-deriving opportunistic pathogen (Li et al., 2004) that has been found in endocarditis (Turnier et al., 2009) and cerebral abscess (Thiagarajan et al., 2016). Reportedly, in neutropenic patients, *S. oralis* induces sepsis (Penack et al., 2014; Shelburne et al., 2014) and inflicts death (Cornely and Schirmacher, 2001). Thus, the mechanisms of *S. oralis*-oriented abnormal coagulation to reduce the mortality need to be deduced. The overwhelming host response can progress to disseminated intravascular coagulation (DIC) by upregulating the activation of the coagulation cascade (Fourrier et al., 1992). It is characterized by excessive activated coagulation systems and suppressed fibrinolytic systems and is known to contribute to the formation of microthrombus, which leads to multiorgan failure (Vervloet et al., 1998). In this process, several coagulation factors patriciate in both classic clotting pathways (Amaral et al., 2004). In addition, most of the coagulation factors in coagulation cascade are generated by hepatocytes. Blood coagulation factor VII (FVII) and factor XII (FXII) are the key enzymes in the extrinsic and intrinsic coagulation cascades (Furie and Furie, 1988). When interacted with tissue factor (TF), FVII is converted to the active state (FVIIa), forms TF–FVIIa complex, and initiates the downstream extrinsic clotting cascade (Bogdanov et al., 2003). In the case of the intrinsic pathway of coagulation, FXII initiates the activation of the blood coagulation zymogen FXI and leads to the formation of factor IXa (Schmaier, 2008). Thus, further exploration of FVII and FXII may provide an in-depth insight into the aberrant coagulation in sepsis.

The circadian clock genes in mammals are endogenous oscillators, containing interlocked and negative feedback loops (Takahashi et al., 2008; Duong et al., 2011). These loops are basic helix-loop-helix-PER-ARNT-SIM (PAS) transcriptional activators *BMAL1* and *CLOCK, NPAS2* in addition to Period (*Per1* and *Per2*), and cryptochrome (*Cry1* and *Cry2*) genes (Koike et al., 2012). Among these genes, *BMAL1* is a non-redundant and an essential component of circadian clock genes for maintaining normal physiology and behaviors (Bunger et al., 2000). Additionally, deficiency of *BMAL1* in mice causes an imbalance in the coagulation mechanism and has potential for blood thrombin generation (Ninivaggi et al., 2014; Hemmeryckx et al., 2019), while other circadian clock genes such as *CLOCK* (Ohkura et al., 2006; Cheng et al., 2011), *Per2* (Oishi et al., 2009), and *Cry1/2* (Ohkura et al., 2006) participate in hemostasis via regulation of fibrinolytic systems rather than coagulation systems. Nevertheless, the mechanisms underlying the functional role of *BMAL1* in affecting coagulation in bacterial infection, such as *S. oralis*, remain largely obscure.

In this study, we demonstrated that *S. oralis* downregulates the expression of *BMAL1* and then disrupts the biosynthesis of coagulation factors. As a transcription factor, *BMAL1* regulates the coagulation genes through the E-box elements. For the first time, this study demonstrated the correlation between *S. oralis* and *BMAL1*. The formation of microthrombus in hematosepsis is related to the balance of abnormal coagulation factors from hepatocyte due to the decline in *BMAL1*. Therefore, recovering *BMAL1* expression might be a promising strategy to inhibit abnormal coagulation for reducing the formation of microthrombus and lowering the mortality.

## Materials and Methods

### Cell Culture and *S. oralis* Infection

HepG2 cells were purchased from ATCC (HB-8065, USA) and cultured in Dulbecco's minimum essential media (DMEM) supplemented with 10% fetal bovine serum (FBS), 100 U/mL penicillin, and 100 μg/mL streptomycin in an atmosphere of 5% CO_2_ at 37 °C. At 70–80% confluency, the cells were trypsinized and challenged by *S. oralis* with a multiplicity of infection (MOI)=50 for 24 h.

### Animals

C57BL/6J mice were bought from the Beijing HFK Bioscience Co. Ltd (Beijing, China) and grouped randomly into either LD12:12 or jet-lagged. Homozygous BMAL1-deficiency (*Bmal1*^−/−^) mice in the C57BL/6J background were produced by breeding heterozygous BMAL1-deficiency mating pairs (*Bmal1*^+/−^), which was a kind gift from Dr. Ying Xu (Soochow University, Jiangsu, China). T BMAL1-deficiency was confirmed by Western blot as described (Bunger et al., 2000).

### RNA Isolation and Reverse Transcription-Quantitative Polymerase Chain Reaction (RT-qPCR)

Total RNA was extracted on ice using TRIzol (Vazyme, China), according to the manufacturer's protocols. cDNA synthesis was performed with reverse transcription reagent (Vazyme). Real-time RT-PCR was performed using SYBR Green PCR protocol on an ABI 7300 real-time PCR system (Applied Biosystems, Carlsbad, CA, USA). Relative mRNA expression of the target genes was normalized against that of *GAPDH* using the 2^−ΔΔ*Ct*^ method, and presented as mean±SD of replicates. The primers used for amplification are listed in Supplementary Table 1.

### Western Blot Analysis

Cells were lysed on ice by modified RIPA buffer (P0013B; Beyotime, Shanghai, China) to obtain total protein extract that was quantitated by BCA protein Assay kit (P0010; Beyotime, Shanghai, China), and resolved by 10% sodium dodecyl sulfate-polyacrylamide gel electrophoresis (SDS-PAGE). Subsequently, the proteins were transferred to polyvinylidene fluoride (PVDF) membranes that were then blocked in 5% milk diluted with TBST for 1 h at room temperature, probed with primary antibodies, such as BMAL1 (Abcam, 1:1000), CLOCK (Abcam, 1:1000), PER2 (Abcam, 1:1000), CRY2 (Abcam, 1:1000), GAPDH (Santa Cruz Biotechnology Inc., 1:10000), FVII (Santa Cruz Biotechnology Inc., 1:1000), and FXII (Santa Cruz Biotechnology Inc., 1:500), at 4°C overnight. Then, the membranes were incubated with a secondary goat anti-rabbit antibody (1:2000) on the following day for 1 h at room temperature. ECL enhanced chemiluminescence substrate kit (Millipore) was used for imaging and quantitation of the immunoreactive bands by the Image J software.

### Immunohistochemistry (IHC)

Liver tissues were fixed with 4% paraformaldehyde in phosphate-buffered saline (PBS) overnight and sliced into 5-μm-thick paraffin-embedded sections. After deparaffinization and rehydration, antigen retrieval was performed by boiling the sections in citrate antigen retrieval solution (P0081; Beyotime) for 15 min. Next, the endogenous peroxidase activity of the samples was blocked by 3% H_2_O_2_ for 15 min, followed by pre-blocking with 5% BSA (A1933; Sigma–Aldrich) for 1 h at room temperature. The levels of proteins were investigated in liver tissues using primary antibodies against BMAL1 (Abcam, 1:800), FVII (Santa Cruz Biotechnology Inc., 1:400), and FXII (Santa Cruz Biotechnology Inc., 1:400) using a Vectastain ABC kit (Vector Laboratories, Burlingame, CA, USA), followed by the DAB Substrate kit (Vector Laboratories). All protocols were followed according to the manufacturer's instructions.

### Immunofluorescence (IF)

Cells were plated at a density of 1 × 10^5^ cells/well in 24-well plates and then grown on glass coverslips for 12 h and infected with *S. oralis*, followed by staining with 5 mM SYTO® Green-Fluorescent Nucleic Acid Stains (Thermo Fisher, USA) for 30 min. After gentle washing for three times with PBS, the cells were fixed for 10 min in 4% paraformaldehyde (wt/vol). The cells were pre-blocked with 5% BSA (A1933; Sigma–Aldrich), labeled with TRITC Phalloidin (Yeasen, 1 μg/mL), and incubated with anti-rabbit Alexa Fluor 594 (Invitrogen) secondary antibodies (1:300) for 1 h at room temperature. The cell nuclei were stained with by DAPI dihydrochloride staining solution. Images were collected using a confocal microscope (SP2-AOBS Leica Microsystems).

### Enzyme-Linked Immunosorbent Assay (ELISA)

The concentrations of mouse plasma FVII (Cusabio, China) and mouse plasma FXII (Cusabio) were measured using ELISA kits according to the manufacturer's instructions. HepG2 cells were seeded overnight in 1% FBS-DMEM in six-well plates (6 × 10^6^/well) and infected with *S. oralis* in 100 U/mL penicillin and 100 μg/mL streptomycin for 24 h (MOI=50). Then, cell-free-culture supernatants were collected and analyzed for FVII or FXII content by a specific ELISA (), according to the manufacturer's recommendations. The cells without infection are similar to infected cells. Briefly, the standard protein was solubilized and diluted to 20, 10, 5, 2.5, 1.25, 0.625, and 0.312 ng/mL for constructing a standard curve. The diluted samples were added to the reaction plate and incubated at 37°C for 2 h. The wells were washed five times, and the biotin primary antibody was added, and the reaction was incubated at 37°C for 1 h. Subsequently, horseradish peroxidase (HRP)-avidin was added and incubated at 37°C for 1 h, followed by the addition of a substrate chromogenic solution that was incubated at 37°C for 30 min. Finally, the reaction terminator and OD_450_ were measured.

### Fluorescence *in situ* Hybridization (FISH)

The following probe was used for FISH: *S. oralis* (probe sequences targeted 16S ribosomal-RNA (rRNA) genes; 6FAM-CTCCTACGGGAGGCAGCAGTAGGGA-BHQ-3; fluorescence emission maximum at 900 nM). As mentioned above, the liver sample was fixed in 4% paraformaldehyde for 2 h at room temperature and stored in 96% ethanol. Then, the paraffin-embedded sections (5-μm-thick) were incubated in hybridization buffer (100 mM Tris-HCl pH 7.2, 0.9 M NaCl, 0.1% SDS) containing 8 ng/mL of the *S. oralis* probe overnight at 37°C. Slides were washed 3 times for 15 min in prewarmed (37°C) hybridization buffer, following the labeling of the cell nucleus by DAPI. Images were collected using a confocal microscope (SP2-AOBS Leica Microsystems).

### *In vivo* Imaging of Animals

For *in vivo* imaging, mice (LD12:12 and jet-lagged) were injected 10^9^ fluorescence-labeled bacteria through the tail vein for 6 h prior to imaging, as described previously. Subsequently, the animals were euthanized with sodium pentobarbital, the hairs were wiped off, and the organs were removed and placed on plates. The fluorescence was detected at 490 nm (*in-vivo* FX PRO BRUKER). The images were analyzed using Bruker software. All procedures were performed using a standard protocol.

### Coagulation Array

A volume of 1.8 mL heart blood was collected in a blood collection tube containing 0.2 mL of 3.2% sodium citrate anticoagulant (blood volume <1.8 mL was prepared according to the ratio of blood to anticoagulant 1:9) and mixed gently. The plasma was separated at 3,000 rpm for 15 min. The prothrombin time (PT), activated partial thromboplastin time (APTT), fibrinogen time, and fibrinogen concentration assays were conducted using a specific kit (Hanlisisw, China). All assays were carried out with an automatic coagulator (BE, Germany).

### Bacterial Strains and Culture Conditions

The strain used for research: *S. oralis* from ATCC. Brain Heart Infusion (BHI) medium (Becton, Dickinson and Company, USA) was used for bacterial growth under aerobic conditions for 24–48 h at 37°C in order to reach the exponential growth phase. Subsequently, 10-fold serial dilutions of each bacterium were performed in PBS to achieve concentrations of ~1 × 10^8^ CFU/mL. Specific primers were designed for *S. oralis* (forward primer: CAACGATACATAGCCGACCTGAG; reverse primer: TCCATTGCCGAAGATTCC).

### Chromatin Immunoprecipitation Assay (ChIP)

Chromatin immunoprecipitation assays were conducted using chromatin immunoprecipitation kit (Millipore), according to the manufacturer's protocol. Briefly, HEPG2 cells were fixed in 1% formaldehyde, cross-linked with glycine, and lysed. Cell lysates were sonicated into fragments and precleared with Protein A/G Agarose beads for 1 h. After centrifugation (1,000 rpm × 1 min at 4°C), 10% of the supernatants were stored as input, and the remaining supernatants were divided into two parts, one for detection by BMAL1 antibody and the remaining for IgG reactivity (negative control). The DNA was purified in a final volume of 50 mL and subjected to PCR. Specific primers were designed for FVII promoter (forward primer: GACCGTCTACCCCAGTGTTT; reverse primer: CGGCAGTCCACGTCATTTC).

### Luciferase Reporter Assay

*Bmal1*-overexpression and *Bmal1*-knockout HepG2 cells were constructed and plated at a density of 5 × 10^5^ cells/well in 12-well plates. These cells were transfected with the wild-type FVII promoter, its deletion mutant, its mutation with mutated BMAL1-binding site, and empty vector (PGL3). Furthermore, firefly luciferase reporter vectors (100 ng) and Renilla luciferase reporter vectors pRL-SV40 (10 ng; Promega, USA) were transfected into cells using Lipofectamine™ 3,000 transfection kit (Thermo Fisher, USA) for 48 h. Subsequently, firefly and Renilla luciferase activity was measured using the Dual-Luciferase Reporter Assay System (Promega). The data were normalized by the firefly/Renilla ratio.

### Transformation of *S. oralis*

Briefly, mid-logarithmic phase cultures of *S. oralis* were diluted 1:20 in complete medium (Brain Heart Infusion, Becton, Dickinson, and Co.) containing 1 mM calcium chloride, 0.2% BSA (Sigma Aldrich Ltd, Dorset, UK) and 100 ng/mL competence-stimulating peptide 1 (CSP1; Mimotopes, Clayton, Victoria, Australia). The transformation reactions were incubated for 90 s at 42 °C and placed on ice for 2 min. Then, the sensory bacteria were transferred to 6-well plate with ampicillin for 16 h. The primers were designed for *GFP* (forward primer: GACCGTCTACCCCAGTGTTT; reverse primer: CGGCAGTCCACGTCATTTC).

### Statistical Analysis

All the statistical analyses were performed with GraphPad Prism Software and based on normally distributed datasets with equal variance (Bartlett's test). The investigators were not blinded during the experiments or outcome assessment. The data points were presented as mean ± SD values unless otherwise stated. Data were inferred as statistically significant if *P*-values were <0.05. Significance between two groups was determined by independent Student's *t*-test. For multiple comparisons, one-way analysis of variance (ANOVA) was used with Tukey's multiple comparisons *post-hoc* test unless stated otherwise.

## Result

### *S. oralis* Reduced BMAL1 Expression and Disrupted the Biosynthesis of Coagulation Factors *in vitro*

**Figure 1:**
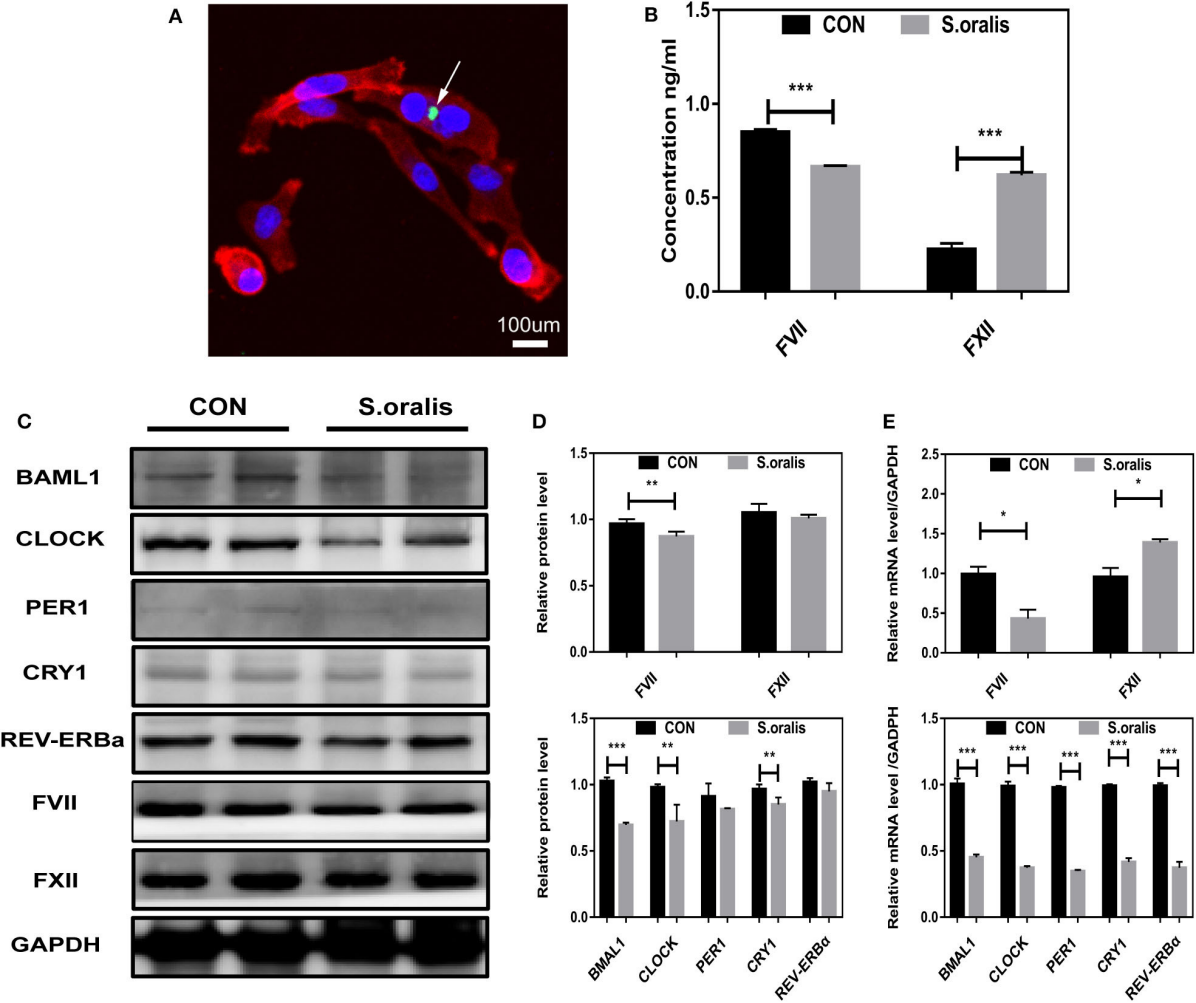
*S. oralis* reduced BMAL1 expression and disrupted the biosynthesis of coagulation factors *in vitro*. **(A)** Confocal images for HepG2 cells infected with *S. oralis* for 30 min, stained for DAPI (blue), F-actin (red), and *S. oralis* (green). The white arrow represents bacteria invading into cells. Scale bars, 100 μm. **(B)** Relative supernatant concentration of coagulation factors VII and XII in HepG2 cells infected with *S. oralis* for 48 h as compared to the uninfected group (CON). **(C,D)** Western blot analysis of circadian clock genes and coagulation factors, and the relative values of densitometry analysis. **(E)** qRT-PCR analysis of circadian clock genes and coagulation factors. The experiments were performed three times independently. *P < 0.05; **P < 0.01; ***P < 0.005.

α-hemolytic *Streptococcus*, such as *S. oralis*, exhibits strong pathopoiesia either by generating various enzymes and exotoxin indirectly or invading into a host directly. In order to elucidate how *S. oralis* interacts with hepatocytes, nucleic acid dye-treated bacteria had infected cells in MOI=10,000 for 30 min, which showed that *S. oralis* directly invaded into the cells (Figure 1A). Owning to the lack of elements initiating coagulation, the clotting factors were not consumed, and hence, it was found that FVII slightly decreased, and FXII increased significantly in the cellular supernatant of infected HepG2 (Figure 1B). To explore the correlation between *S. oralis* infection and circadian clock genes in hepatocytes, cells were treated with *S. oralis*, and the total protein and mRNA were extracted. Consequently, *BMAL1* expression was significantly decreased as compared to the other circadian clock genes, and it was found that the coagulation factor FVII decreased slightly, while the FXII did not alter (Figures 1C,D). In another cohort of cells with the same treatment, the expression of *BMAL1* and other circadian clock genes was lower in *S. oralis* infection group, and obvious upregulation of FVII and mild downregulation of FXII was detected at the transcriptional level (Figure 1E).

### *S. oralis* Reduced BMAL1 Expression and Disrupted the Biosynthesis of Coagulation Factors *in vivo*

**Figure 2:**
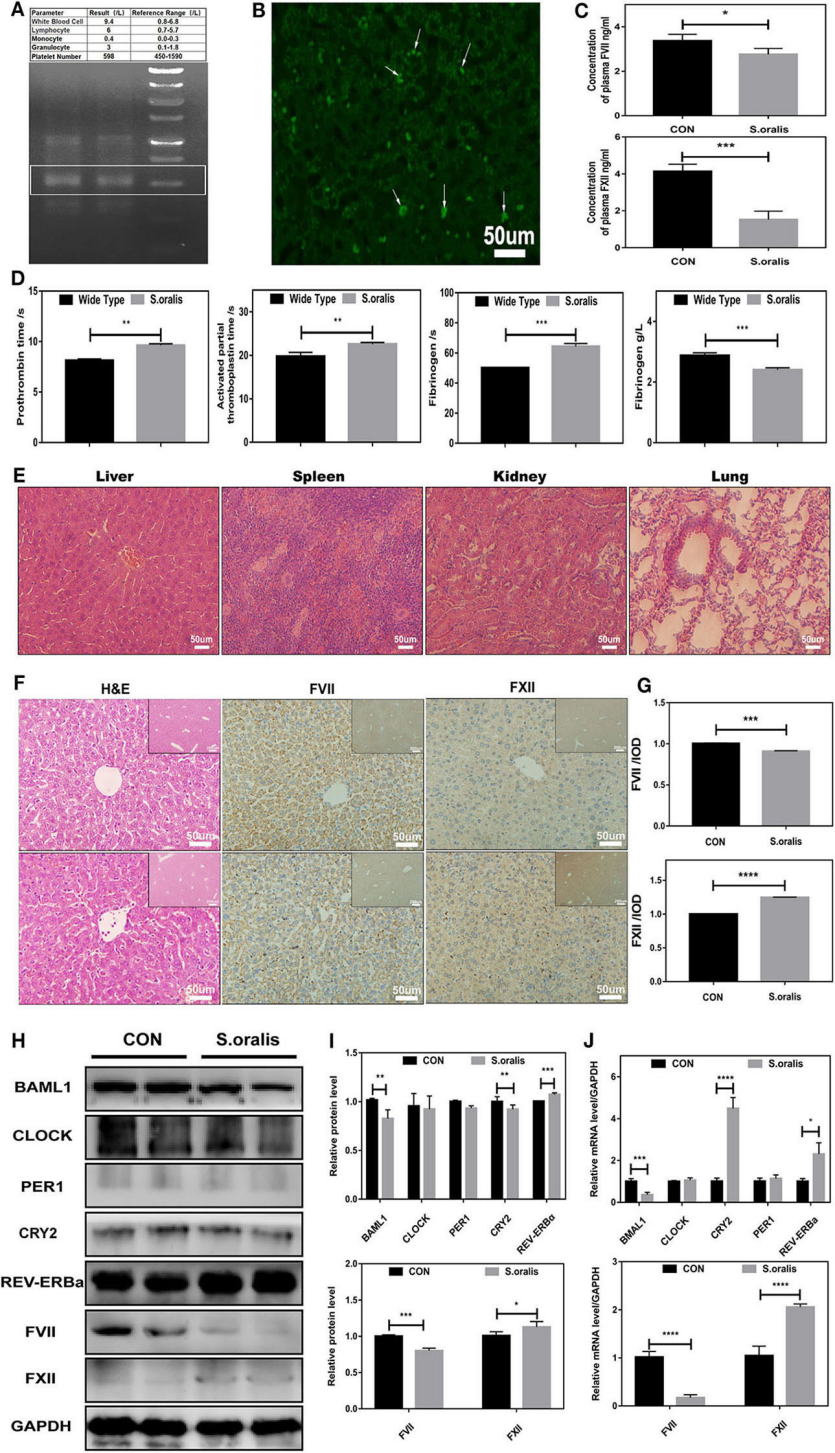
*S. oralis* reduced BMAL1 expression and disrupted the biosynthesis of coagulation factors *in vivo*. Every mouse was infected by *S. oralis* in about 1 × 10^8^ CFU (colony-forming units) for 24 h. **(A)** The upper part for the blood routine test of the infected mouse, the lower part for DNA extraction of infected mouse livers. Special primers were designed for *S. oralis*. **(B)** FISH for the detection of *S. oralis* in infected mouse livers. Special primers were designed for *S. oralis*. Scale bars, 50 mm. **(C)** Plasma concentration of coagulation factors VII and XII in the infected group as compared to the control group. **(D)** Routine coagulation array for mice in infected and uninfected groups. **(E)** H&E staining for liver, spleen, kidney, and lung of infected mice. **(F,G)** IHC staining for coagulation factors VII and XII of the liver and the relative values of IHC optical density. **(H,I)** Western blot analysis of circadian clock genes and coagulation factors, and the relative values of densitometry analysis of hepatocytes. **(J)** qRT-PCR analysis of circadian clock genes and coagulation factors. The experiments were performed three times independently. *P < 0.05; **P < 0.01 ***P < 0.005; ****P < 0.001.

Correspondingly, to explore whether *S. oralis* modified the circadian clock genes and coagulation factor levels *in vivo*, another series of studies were carried out. The mice were injected with *S. oralis* into the tail vein. After the mice were sacrificed, the whole blood was collected for a routine examination, and it was found that the neutrophils increased significantly. In addition, the total DNA of the liver and spleen was extracted and amplified to detect the bacteria in these organs using *S. oralis*-specific primers (Figure 2A). To further consolidate the presence of bacteria in the liver, FISH was conducted, which showed green EGFP-labeled-bacterium in the liver, and many of them had invaded into the hepatocytes (Figure 2B). This phenomenon might suggest that *S. oralis* plays a functional role both directly and indirectly. The remaining of the whole blood was collected for coagulation arrays and ELISA. It was observed that the level of both plasma coagulation factors, FVII and FXII, was downregulated (Figure 2C). Additionally, it was found that PT, APTT, and fibrinogen reaction time were prolonged and plasma fibrinogen was declined (Figure 2D). In sepsis, bacteria can activate both intrinsic and extrinsic coagulation by the cytokine response. The serial sections of liver, spleen, kidney and lung stained with hematoxylin and eosin (H&E) indicated inflammation after infection (Figure 2E). Moreover, most of the coagulation factors, including FVII and FXII were generated in the liver. To investigate the correlation between *S. oralis* infection and coagulation factors, serial sections of the liver tissues were stained with H&E, and histochemistry was conducted (Figure 2F). *S. oralis* infection declined the expression of FVII expression, while that of FXII was elevated (Figure 2G). In addition, the livers of mice were collected and the total proteins were extracted. The results showed that BMAL1 expression was significantly decreased as compared to that of the other genes; also, an obvious decline was detected in FVII and a mild elevation of FXII as compared to the uninfected group (Figures 2H,I). On the other hand, *BAML1* level was also decreased, while the mRNA expression of *REV-ERBa* and *CRY1* was elevated, *FVII* was significantly downregulated, and *FXII* was markedly upregulated (Figure 2J). Therefore, we confirmed that *S. oralis* infection declined the level of FVII and BMAL1 while that of FXII was elevated at both protein and transcriptional levels *in vivo*.

### Downregulation of FVII and Upregulation of FXII in *Bmal1*^-/-^ Mice

**Figure 3:**
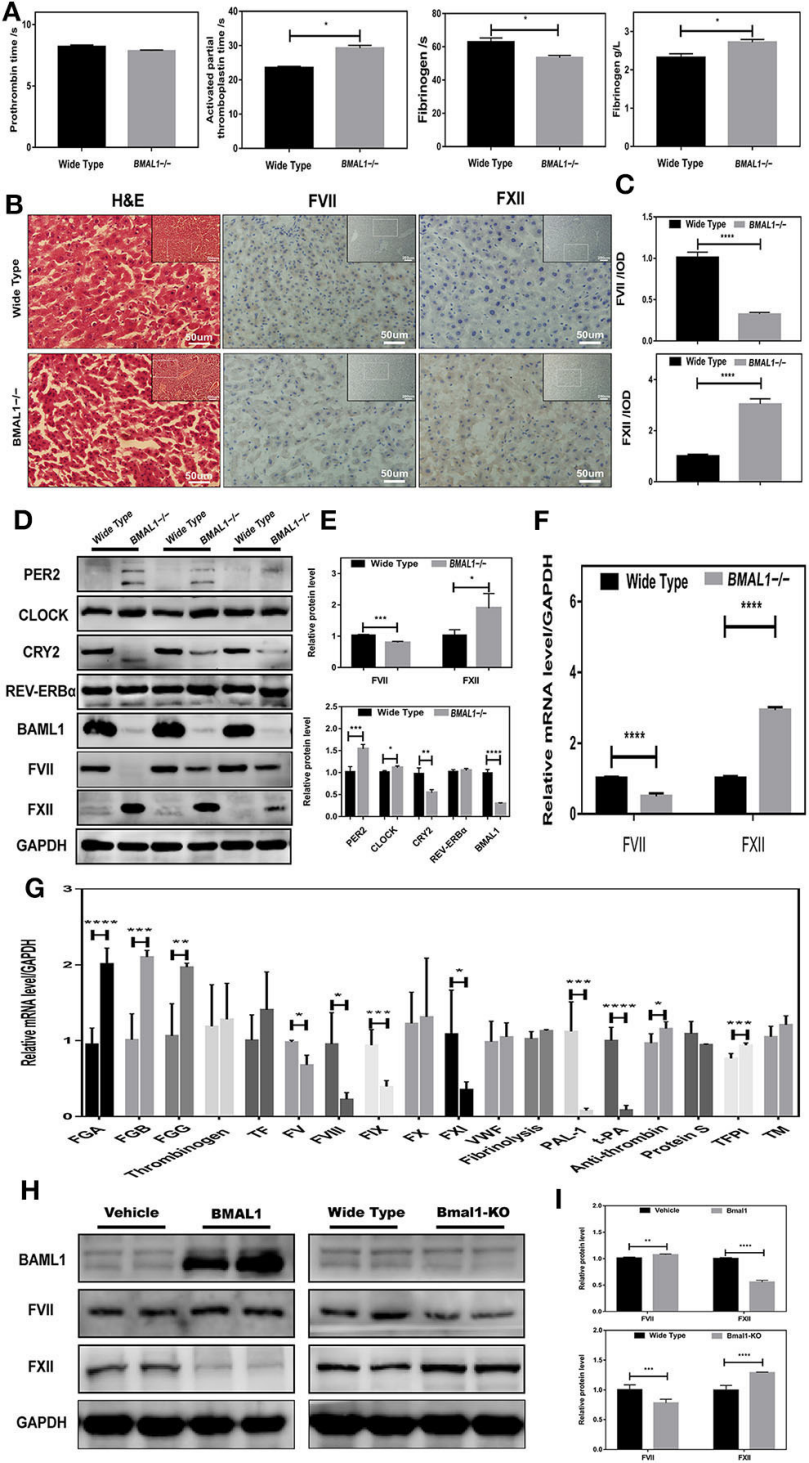
Disrupted coagulation factors VII and XII in Bmal1^−/−^ mice. **(A)** Routine coagulation array for whole blood in *Bmal1*^−/−^ vs. WT mice (3/group). **(B,C)** H&E and IHC staining for coagulation factors VII and XII in slices of livers, and the relative values of IHC optical density. **(D–G)** Western blot analysis and qRT-PCR analysis of circadian clock genes and coagulation factors in mouse liver. **(H,I)** Western blot analysis of coagulation factors VII and XII in *Bmal1*-overexpression and Bmal1-knockout HepG2 cells. The experiments were performed three times independently. *P < 0.05; **P < 0.01; ***P < 0.005; ****P < 0.001.

To confirm the role of BMAL1 in coagulation factor biosynthesis, we constructed BMAL1-deficiency (*Bmal1*^−/−^) mice, and the genotypes were verified by Western blot (Figure 2D). Interestingly, we found that BMAL1 had two-aspect functions in the coagulation process. The blood coagulation arrays showed that PT was slightly deduced while APTT was significantly prolonged with a huge reduction in these fibrinogen reaction time owning to elevated plasma fibrinogen in *Bmal1*^−/−^ mice (Figure 3A). FVII and FXII were the key enzymes that initiated the extrinsic and intrinsic coagulation cascade, respectively. Therefore, the serial sections of the liver tissues were stained with H&E, and histochemistry was performed (Figure 3B). The result showed that FVII approximately decreased to a third, and FXII increased nearly four-fold in *Bmal1*^−/−^ mice (Figure 3C). To further verify the correlation between BMAL1 and the biosynthesis of coagulation factors, the total protein of the liver tissues was extracted. This phenomenon was similar to that FVII decreased significantly while FXII increased by three-fold in *Bmal1*^−/−^ mice (Figures 3D,E). In addition, that the expression of the coagulation factor was altered at the mRNA level, which was synchronous with the changes in the protein level, indicating that BMAL1 was likely to act as a transcription factor to regulate the biosynthesis of coagulation factors (Figure 3F). Although coagulation factors FVII and FXII played major roles in coagulation cascades, other coagulation factors, such as vWF, also played a significant role. Therefore, we analyzed the expression of other coagulation factors (Figure 3G). To investigate the effect of BAML1 on the biosynthesis of coagulation factors *in vitro, Bmal1*-overexpression or *BAML1*-knockout was used (Figure 3H), which showed that FVII expression increased mildly and FXII significantly decreased in *Bmal1*-overexpressing cells. However, a contrast result was observed in *BAML1*-knockout cells (Figure 3I). It was also confirmed that the deficiency of BMAL1 negatively regulated FVII expression but positively regulated FXII.

### BMAL1 Regulates Coagulation Factors VII in a Direct Way

**Figure 4:**
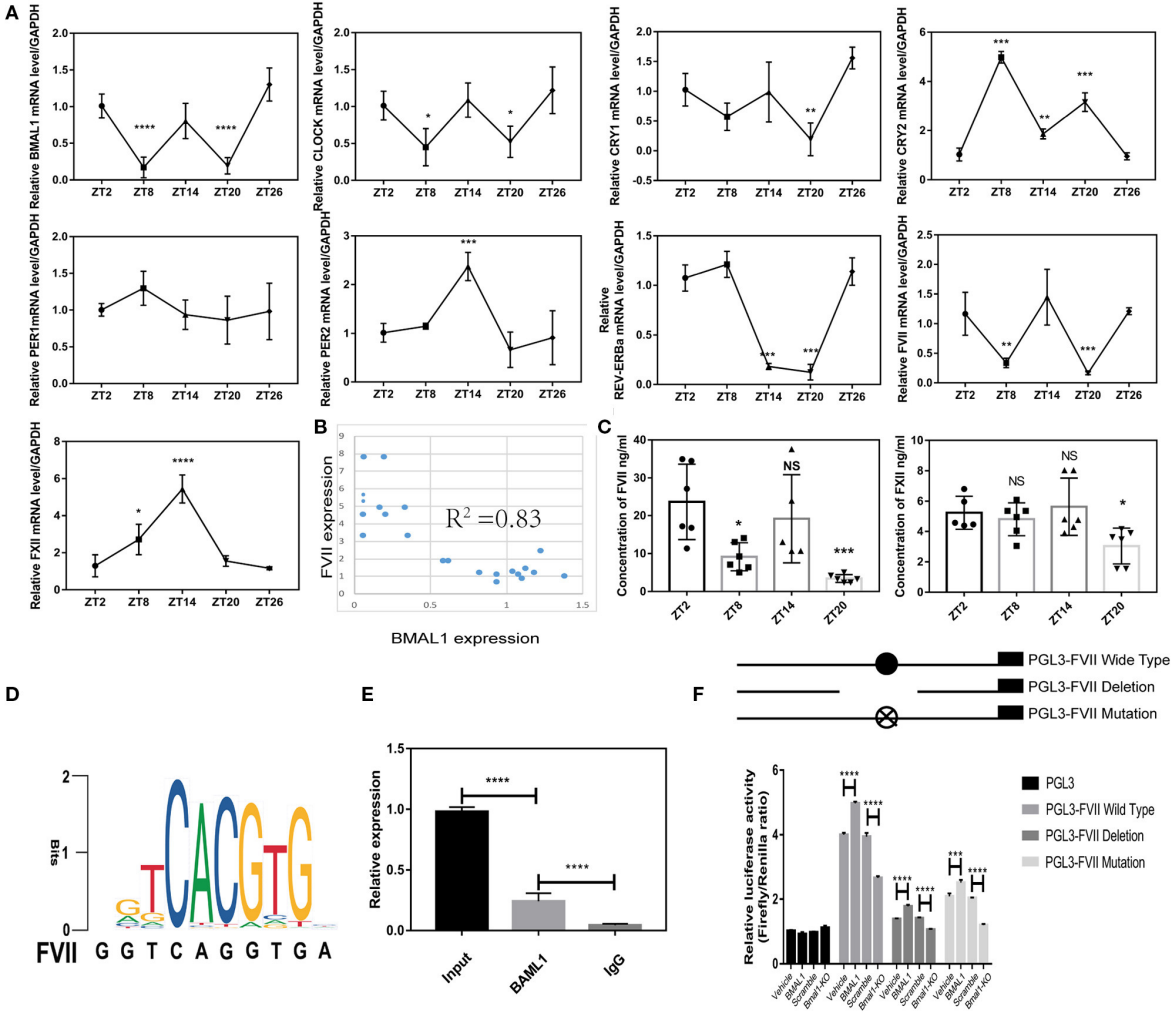
BMAL1 regulates coagulation factors VII in a direct way. **(A)** Relative expression of circadian clock genes and coagulation factors at different time points (4 mice/each point). **(B)** Correlation analysis between BMAL1 and FVII. **(C)** Plasma concentration of coagulation factors VII and XII at each time point. **(D)** Motif sequence signature of BMAL1 in the promoter region of coagulation factor FVII. **(E)** ChIP assays were performed using an antibody against BMAL1, and IgG as negative controls. Specific primers were designed for FVII promoter. **(F)** Luciferase reporter assays were performed to measure the activities of WT FVII promoter (PGL3- FVII WT), its deletion mutant (PGL3- FVII deletion), its mutant (PGL3- FVII mutation) with mutated BMAL1-binding site and empty vector (PGL3) in *Bmal1*-overexpression and *Bmal1*-knockout HepG2 cells. The experiments were performed three times independently. *P < 0.05; **P < 0.01; ***P < 0.005; ****P < 0.001.

Firstly, we found that *FVII* mRNA level showed 12-h circadian changes and reached nadir around ZT8 and ZT20, but FXII did not exhibit circadian changes; several circadian clock genes in the liver showed obvious rhythmicity, and *BMAL1* mRNA expression was synchronous with FVII expression by hitting valleys around ZT8 and ZT20 (Figure 4A). Furthermore, the correlation between circadian clock genes and FVII showed that the expression of *BMAL1* was strongly associated with that of *FVII* (Figure 4B). To further verify the rhythmicity of FVII expression, we measured the plasma coagulation factor at different time points. Strikingly, FVII expression reached nadir around ZT8 and ZT20, while that of FXII lacked circadian changes (Figure 4C). Typically, BMAL1 acts as a transcription factor that forms a heterodimer with CLOCK to activate the expression of the downstream genes, such as E-box-controlled genes PER1 and CRY1 (Duong et al., 2011). Therefore, it could be deduced that BMAL1 regulated *FVII* through transcription factor binding sites (Figures 4D,E).

Low activity of *FVII* promoter could be found in cells transfected with both the *FVII* promoter mutants with *Bmal1* binding site and the *FVII* promoter deletion mutant. To confirm the role of BMAL1, firefly luciferase reporter vectors were transfected into *Bmal1*-overexpressing and *Bmal1*-knockout cells. Next, we found that the activity of *FVII* promoter was enhanced in *Bmal1*-overexpressing cells, while it was declined in *Bmal1*-knockout cells. Together, these results suggested that BMAL1 directly regulates the expression of *FVII* in a positive way (Figure 4F).

### Circadian Rhythm Disruption-Driven Coagulation Prevents Extravasation of *S. oralis*

**Figure 5:**
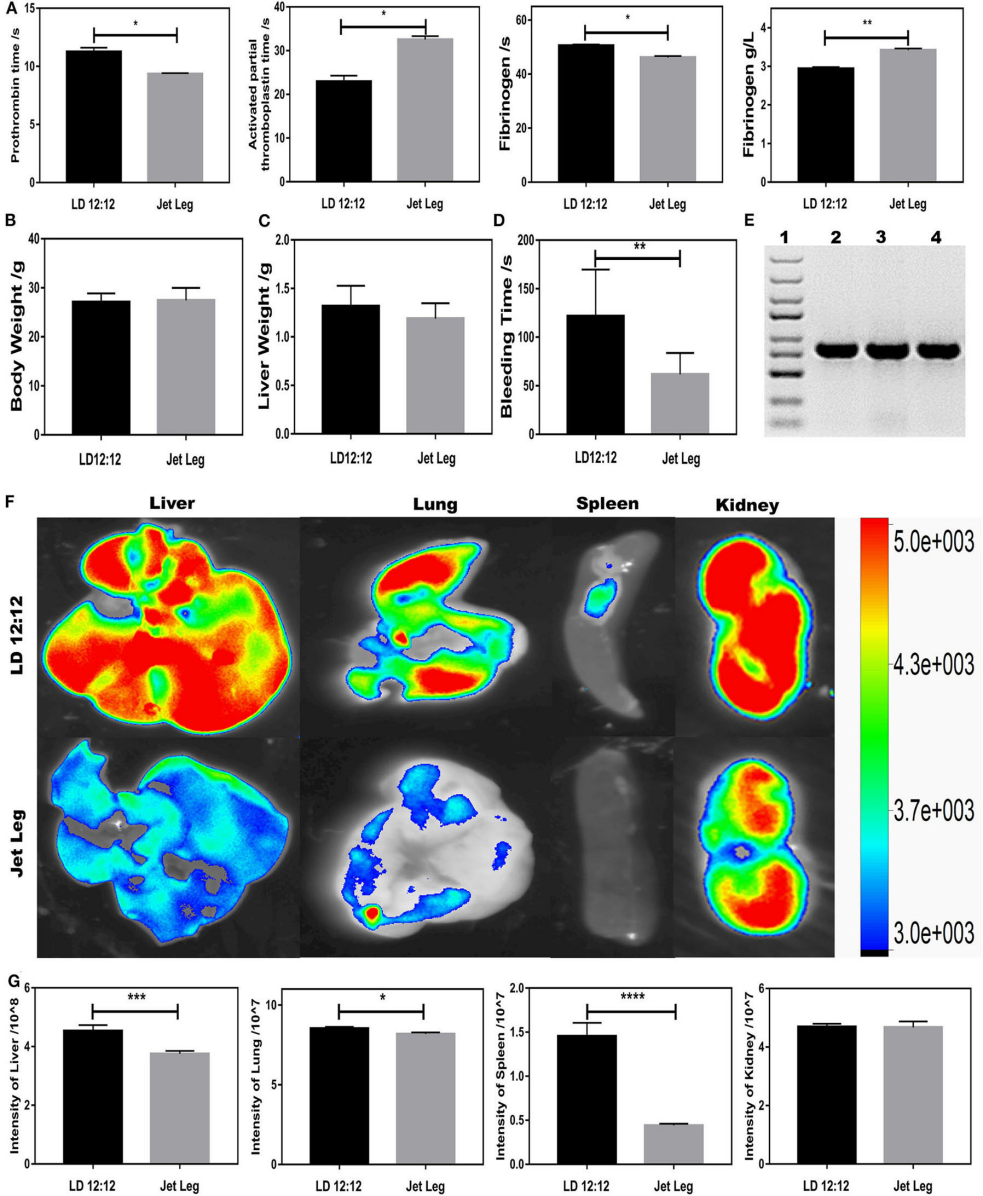
Circadian rhythm disruption-driven coagulation prevents extravasation of *S. oralis*. **(A)** Routine coagulation array (4 mice/group) for whole blood in LD12:12 vs. jet-lag mice at ZT12. **(B,C)** Bodyweight and liver weight of mice in LD12:12 vs. jet-lag at ZT12. **(D)** Tail bleeding time of mice. **(E)** DNA extraction of EGFP-labeled *S. oralis*. Specific primers for GFP. **(F,G)** Imaging of mice in LD12:12 vs. jet-lag mice at ZT2 *in vivo* (4 mice/group). *P < 0.05; **P < 0.01; ***P < 0.005; ****P < 0.001.

Since BMAL1 plays a key role in circadian rhythm, investigating the circadian rhythm disruption-driven coagulation is crucial for unveiling the role of BMAL1 in coagulation. To further explore the roles of circadian clock gene *BMAL1* in coagulation, a jet-lagged mouse model was established to observe the impact of circadian rhythm disruption on the impairment of the liver ossification process. All mice were maintained under 12/12-h light/dark cycles with the light on from 8:00 a.m. (Zeitgeber time 0, ZT0) to 8:00 p.m. (ZT12) and fed antibiotic-free food and water *ad libitum*. In the experiment, C57BL/6J mice were placed on a previously described jet-lagged schedule with 8-h light advanced every 2–3 days for 12 weeks, which mimicked the circadian disturbance that humans undergo during shift work (Zhao et al., 2017). PT was mildly shortened, while APTT was significantly prolonged, and the fibrinogen reaction time was shortened owing to elevated plasma fibrinogen in jet-lagged mice (Figure 5A). To eliminate the impact of body weight and liver weight, measurement experiments were essential, which seemed to have no impact on the coagulation (Figures 5B,C). However, jet-lag seemed to induce a slight decline in BT, suggesting that circadian rhythm disruption-driven downregulation of BMAL1 might be conducive to thrombus formation (Figure 5D). In order to elucidate whether circadian rhythm disruption-driven coagulation prevents extravasation of *S. oralis*, EGFP-labeled *S. oralis* was constructed and confirmed by PCR amplification (Figure 5E). We found that the number of bacteria invaded into the liver was less than that in the LD12:12 group due to disruption-driven coagulation, which prevents the invasion of EGFP-labeled *S. oralis*. Intriguingly, a similar phenomenon was observed in the lung and spleen, and that the number of bacteria invaded into the kidneys was large in quantity in both the jet-lagged and LD12:12 groups because of abundant blood vessels (Figures 5F,G).

## Discussion

To gain new insight into the mechanism underlying the influence of the circadian gene *BMAL1* on coagulation factors in bacterial infectious liver disease, we performed the experiment described above and showed that the level of BMAL1 in hepatocytes was downregulated in the *S. oralis* infection group, leading to the downregulation of coagulation factor VII and the upregulation of coagulation factor XII. Similar results were found *in vitro*. *Bmal1* overexpression significantly increased the coagulation factor VII while it decreased the level of coagulation factor XII in HEPG2 cell line. According to these findings, circadian gene *BMAL1* plays a critical role in coagulation by regulating the biogenesis of clotting factors in *S. oralis* infection.

Reportedly, the gut homeostatic microbiome is necessary for normal oscillations in both intestine and liver, which is effectuated via circadian genes, and participating in diurnal fluctuations of host physiology, disease susceptibility, lipid absorption, and sleep rhythms (Miyazaki et al., 2014; Thaiss et al., 2016; Kuang et al., 2019). The courses of bacterial invasion into the host include bacterial adsorption on the body surface, invading tissues or cells, growing and breeding, producing toxins, spreading, and resisting a series of defensive functions of the host. Colonized *S. oralis* derived from blood bacteria was found in the liver and was also found to be invading into hepatocytes (Figures 1A, 2A). Herein, we observed that the downregulation of BMAL1 contributed to the secretion of FXII without the upregulation of FXII protein level *in vitro* (Figure 1B). Consistent with the current findings, some studies have shown that the deficiency of BMAL1 upregulates the secretion of many biochemical parameters by restraining their repressors, such as CXCL5 (Gibbs et al., 2014) and interleukin-1β (Luyts et al., 2014). Furthermore, the interaction between *Bmal1* of hepatocytes and bacterial infection revealed that the expression of BMAL1 descended at the transcriptional level both *in vitro* and *in vivo* in the microorganism. This finding is in line with the results of the previous studies (Lou et al., 2017; Li et al., 2018; Coiffard et al., 2019). With the infection of *Mycobacterium tuberculosis*, the level of BMAL1 was significantly decreased in both the lung and the spleen (Lou et al., 2017). Conversely, some studies showed a certain promoting effect of BMAL1 on gastric epithelial cells with the infection of *Helicobacter pylori* (Li et al., 2018), which could be attributed to the various roles of BMAL1 in different diseases or the various types of bacteria executing infections in different conditions. The reciprocal role between bacteria and BMAL1 has been reported. The magnitude of response to the pathogenic bacterial challenge under the control of Bmal1 has been established (Bhardwaj et al., 2011; Wang et al., 2011; Gibbs et al., 2012, 2014). However, the mutual role of *Bmal1* and microbiota needs to be investigated in the future.

Circadian rhythm is a regular recurrence of biological processes or activities with an ~24-h cycle. The circadian changes have been detected in human coagulation factors, such as antithrombin, FVII, and FVIII (Kapiotis et al., 1997; Undar et al., 1999; Iversen et al., 2002). In the current study, not only mouse plasma FVII but also FVII in mouse hepatocyte exhibit rhythmicity (Figure 4A). BMAL1 plays a core and elemental role (Bunger et al., 2000). However, other components of circadian clock genes, such as *CLOCK* and *CRY1/2* (Ohkura et al., 2006; Cheng et al., 2011), were largely relevant to the fibrinolytic system rather than the coagulation system. The properties of diurnal rhythm were entirely lost for FVII and antithrombin. Nonetheless, additional evidence on the regulation of coagulation factors needs to be unveiled in *Bmal1*^−/−^ mice. This study showed that FVII level was tremendously declined in *Bmal1*^−/−^ mice (Figures 3C,E). Interestingly, FXII level was markedly elevated in *Bmal1*^−/−^ mice (Figures 3C,E). Thus, our findings may be a significant breakthrough that BMAL1 plays a two-sided role in regulating the coagulation process. In the endogenous coagulation pathway, decreased FXI and FXI might explain the prolonged APPT despite the elevated level of FXII in *Bmal1*^−/−^ mice. In the exogenous coagulation pathway, deficiency of *BAML1* might explain the unchanged PT by affecting the activation of coagulation factors despite deduced FVII in *Bmal1*^−/−^ mice (Figure 3G). This displays that the regulation of FVII is complicated and sophisticated. BMAL1 is crucial for the regulation of FVII expression. Low expression of BMAL1 could still be found in *Bmal1*-knockout cells (Figure 3G). These phenomena might explain the low activity of FVII promoter, which could be found in *Bmal1*-knockout cells (Figure 4F). In the future, it might be essential to study the correlation between BMAL1 and coagulation.

The elevated FXII level ascribes the potentially elevated generation of endogenous thrombin larger in BMAL1-deficient mice (Hemmeryckx et al., 2019). Additionally, the level of FGA (Fibrinogen α chain), FGB (Fibrinogen β chain), and FGG (Fibrinogen γ chain) was elevated in *Bmal1*^−/−^ mice in accordance with the elevated plasma fibrinogen in the blood coagulation arrays, which contributed to the formation of the crypto-fibrin network (Figure 3G; Ninivaggi et al., 2014). Although circadian rhythm disruption-driven coagulation prevents the extravasation of *S. oralis*, more macrothrombusus is induced than in the control group, which leads to multiple organ failure on account of deduced BMAL1 expression.

In summary, our results demonstrated that the downregulation of BMAL1 by *S. oralis* infection disrupts these clotting factors in hepatocytes. Therefore, we identified the coagulation factors VII and XII that were largely regulated in *Bmal1*^−/−^ mice. Interestingly, this study on circadian clock genes *BMAL1* might provide a novel view for the generation of microthrombus in hematosepsis induced by *S. oralis*.

## Data Availability Statement

All datasets generated for this study are included in the article/Supplementary Material.

## Ethics Statement

The animal study was reviewed and approved by the Committee of Ethics of Wuhan Union Hospital and the Institutional Research Ethics Committee of Tongji Medical College, Huazhong University of Science and Technology (Wuhan, China). Written informed consent was obtained from the owners for the participation of their animals in this study.

## Author Contributions

LC and SL designed the study and critically revised the manuscript. JN performed the experiments, analyzed the data, and drafted the manuscript. JZ participated in the implementation of the study. SY, YL, and JP collected the data. All authors read and approved the final manuscript.

## Conflict of Interest

The authors declare that the research was conducted in the absence of any commercial or financial relationships that could be construed as a potential conflict of interest.
